# Effects of live and pasteurized forms of *Lactobacillus casei Zhang* on acute kidney injury and chronic renal fibrosis

**DOI:** 10.1007/s42770-024-01491-y

**Published:** 2024-08-26

**Authors:** Xiuru Wang, Mengxia Shi, Chujin Cao, Rui Zeng, Ying Yao

**Affiliations:** 1 Division of Nephrology, Tongji Hospital, Tongji Medical College, Huazhong University of Science and Technology, 1095 Jiefang Avenue, Wuhan, 430030 China; 2 Division of Nutrition, Tongji Hospital, Tongji Medical College, Huazhong University of Science and Technology, 1095 Jiefang Avenue, Wuhan, 430030 China

**Keywords:** *Lactobacillus casei Zhang*, Pasteurized probiotics, Live probiotics, Acute kidney injury, Chronic renal fibrosis

## Abstract

*Lactobacillus casei Zhang* (*Lac.z*), isolated from traditional sour horse milk in Inner Mongolia, can alleviate various diseases and promote health. Our previous studies found that pretreatment with live *Lac.z* (L-*Lac.z*) could significantly attenuate acute kidney injury and delay the progression of chronic renal fibrosis. However, it is unknown whether these effects could be maintained by pasteurized *Lac.z* (P-*Lac.z*). Mouse models of acute kidney injury and chronic renal fibrosis induced by renal bilateral ischemia-reperfusion (BIR) surgery were treated with L-*Lac.z* or P-*Lac.z* by gavage. Serum and kidney samples were collected to analyze the extent of renal injury and fibrosis, and proteomics was used to explore the potential mechanisms underlying the differences in the effects of the two forms of *Lac.z*. The results revealed that treatment with L-*Lac.z* led to a reduction in serum urea nitrogen levels and in less renal tubular injury and subsequent renal fibrosis after BIR-induced renal injury, whereas these effects were not observed in the P-*Lac.z* group. Proteomic analysis revealed 19 up-regulated proteins and 39 down-regulated proteins in the P-*Lac.z* group, and these gene products were associated with growth and stress resistance. The specific nephroprotective effects of L-*Lac.z* may be independent of the interaction of live probiotics with the host.

**Supplementary Information:**

The online version contains supplementary material available at 10.1007/s42770-024-01491-y.

## Introduction

Acute kidney injury (AKI) has become a serious global public health issue with high rates of morbidity and mortality [1]. Epidemiological studies have revealed that approximately 40% of AKI survivors suffer from chronic kidney injury, and 10-20% require ongoing dialysis treatment, with a long-term mortality rate exceeding 30% [2]. AKI is also recognized as an independent risk factor for chronic kidney disease (CKD) [3], while effective strategies to prevent the progression from AKI to CKD are still lacking.

The human gut harbors a complex and dynamic microbiome, commonly referred to as the gut microbiota [4] which has is increasingly being linked to host health and disease [5]. Gut microbial-derived metabolites, including amino acids and fibre ferments such as short-chain fatty acids (SCFAs) and bile acid modification, have profound effects on gut homeostasis and the outcome of kidney disease [6–8]. For example, SCFAs are protective against acute tubular injury in an AKI mouse model [9]. Patients with CKD have lower levels of serum and faecal SCFAs, and when given butyric acid, and butyrate treatment delayed the progression of CKD [10]. However, there is increasing evidence showing that imbalances in the gut microbiota, known as gut dysbiosis, and damage to the gut mucosa are associated with inflammation, oxidative stress and renal injury in a variety of renal diseases [11–14]. The interactions between the gut and the kidney are bidirectional. On the one hand, uremia affects the composition and metabolism of the gut microbiota; on the other hand, uremic toxins, such as p-cresyl sulphite and indoxyl sulphate, originated from microbial metabolism, are increased in the presence of gut dysbiosis. And the disruption of the epithelial barrier increased the host’s exposure to uremic toxins, which, in turn exacerbates renal damage [15]. For example, patients with end stage renal disease (ESRD) develop dysbiosis with an increased abundance of microbiota responsible for synthesizing uremic toxins; and in a rat model of CKD, two species in the gut microbiota, *Eggerthella lenta* and *Fusobacterium nucleatum*, increase the production of uremic toxins and contribute to the severity of kidney disease [6].

Probiotics, live microorganisms administered in sufficient quantities to confer health benefits, have shown promise in improving gut barrier function, controlling the overgrowth of harmful gut bacteria, reducing inflammation, enhancing immune tolerance, and competing for nutrients in the context of CKD [14, 16–22]. The potential mechanisms by which probiotics act focus on two main aspects. On the one hand, intrinsic components of the probiotics interact with the gut microbiota, as in the case of surface piliation of probiotics involved in binding to inflammatory receptors and mediating the intestinal inflammatory response. For example, SpaCBA, surface piliation of Lactobacillus *rhamnosus GG*, can be a promoter of the activation of Toll-like receptor 2-dependent signaling pathways in HEK cells and the regulation of the production of pro-inflammatory and anti-inflammatory cytokines (TNF-α, IL-6, IL-10, and IL-12) in human monocyte-derived dendritic cells, which help us better understand the multiple probiotic effects of *rhamnose GG* in the human gut and kidney diseases [23]. On the other hand, probiotic metabolites interact with the intestinal mucosa or microbiota, or metabolites enter the circulatory system to perform their functions [16, 22]. Probiotics can alter the distribution of bile acids in the intestine and regulate hepatic cholesterol synthesis, which could improve metabolic disorders. And they can either ferment dietary fibre themselves or increase the abundance of intestinal fibre-producing flora to produce SCFAs that bind to G-protein-coupled receptors 41/43 (GPR41/43) in intestinal epithelial cells to inhibit intestinal inflammation, and SCFAs could enter the circulation system to regulate the immune response of the kidney, thereby reducing renal inflammation and fibrosis [24, 25].

*Lactobacillus casei Zhang* (*Lac.z*), derived from homemade koumiss in Inner Mongolia, China, has been demonstrated to play beneficial roles in several diseases, such as regulating cholesterol and preventing liver injury; improving impaired glucose tolerance and preventing type 2 diabetes, and reducing the risk of colon tumors [26–29]. Our previous research found that *Lac.z* could effectively alleviate bilateral ischemia-reperfusion (BIR)-induced AKI, delay the progression of chronic renal fibrosis, and slow the decline of kidney function in individuals with CKD stages, 3–5 [30]. On the one hand, *Lac.z* restored the level of gut bacteroidetes levels, ameliorated AKI-induced gut dysbiosis and attenuated intestinal inflammation and injury. On the other hand, *Lac.z* attenuated renal injury through its metabolites such as SCFAs and nicotinamide: *Lac.z* increased the levels of SCFAs in the serum and kidney, which interacted with SCFA-associated transporters and receptors on the surface of renal tubular epithelial cells and renal macrophages to attenuate the inflammatory response and subsequent fibrosis. Besides, *Lac.z* increased renal nicotinamide levels, which inhibited renal inflammatory cell infiltration.

Although numerous studies have demonstrated the important role of probiotics in ameliorating various diseases, some studies have further investigated whether some probiotics retain their beneficial effects after pasteurization or heat sterilization. For example, both live and pasteurized forms of *Akkermansia muciniphila* could improve high-fat diet-induced (HFD) metabolic disorders, ameliorate HFD/ carbon tetrachloride (CCI4)-induced liver injury, and maintain intestinal barrier integrity by reducing intestinal inflammation [31–33]. And heat-killed *Lactobacillus paracasei Shirota* strain (56–90℃ for 30 min) can produce high TNF-α secretion of stimulated monocytes when administered at higher concentrations. Although our previous study demonstrated that live *Lac.z* ameliorated gut dysbiosis, attenuated AKI and subsequent fibrosis through producing beneficial metabolites, it was unknown whether these beneficial effects could be maintained after pasteurization or heat sterilization. Compared to heat inactivation treatment, pasteurization, with lower temperature (30 min, 70℃), is more likely to maintain the stability of the protein structure of probiotics while inactivating them. Therefore, our study aims to compare the effects of live or pasteurized *Lac.z* on AKI and subsequent chronic renal fibrosis, and to further explore the potential mechanisms underlying the differences in the effects of the two forms of *Lac.z*. through proteomics.

## Materials and methods

### Mice

All mice used in this study were male C57BL/6J mice (8–10 weeks old, weighing 22–25 g) obtained from Biont Biological Technology Co., Ltd, Hubei, China. They were housed in an SPF environment with a 12-hour day and night cycle and had unrestricted access to food and water supply in the Animal Facilities of Tongji hospital experimental animal center. All mice were adaptively fed for one week before experimental use, and animal experiments were reviewed and approved by the Animal Care and Use Committee of Tongji Hospital (TJH-202306010).

### Bacteria preparation and probiotic culture

The live probiotic strain *Lac.z* (L-*Lac.z*) was generously provided by Prof. Zhang (Inner Mongolia Agricultural University, China), as a lyophilized powder with a dilution carrier of dry porous dextrin (which improves the lyophilization survival and storage stability of *Lac.z* [34]) at the total live bacteria concentration of 1 × 10^9 CFUg^− 1^. To obtain the pasteurized *Lac.z* (P-*Lac.z*), the live lyophilized powder was treated at 70℃ for 30 min [35]. To verify the activity of the probiotics after pasteurization, L-*Lac.z* and P-*Lac.z* were propagated in modified Sabouraud’s Agar medium (Hope Bio-Technology, China) anaerobically at 37℃ for 21 h and 96 h. And only the L-*Lac.z* grew in the medium (Supplementary Fig. 1), so the *Lac.z* were inactivated after pasteurization, which means P-*Lac.z* cells lack of viability and physiological activity. For animal experiments, 10 g lyophilized powder of L- *Lac.z* or P- *Lac.z* was reconstituted in 6 mL saline without additives for oral administration. Simultaneously, 10 g placebo (dry porous dextrin) was reconstituted in 6 mL saline without additives as a control for oral administration. The lyophilized powder of L- *Lac.z* and P- *Lac.z* were directly collected for proteomic analysis, and three biological replicates of the samples were prepared in each case.

### Renal bilateral ischemia-reperfusion injury model

Mice were fasted for 8 h prior to surgery and allowed free access to water. After an intraperitoneal injection of 1% pentobarbital sodium solution (0.009 ml/g, Sigma, USA), the mice were anesthetized and secured on a surgical plate, with body temperature maintained between 36.8–37.2℃. Bilateral incisions were made on the back to expose the kidneys, and the renal vascular bundles were clamped using specialized mouse arterial forceps for 30 min before being released. The wounds were then closed when renal blood flow was fully restored. Referring to our previous published studies [30], for the AKI model, mice were pretreated with L-*Lac.z* or P-*Lac.z* (1 × 10^9 CFU per day) by gavage for 28 days, and on day 28, mice were subjected to the BIR surgery, followed by no probiotic intervention, and were killed on day 5 after surgery. For the CKD model, mice were subjected to the BIR surgery, and on day 4 after surgery, mice were treated with L-*Lac.z* or P-*Lac.z* (1 × 10^9 CFU per day) by gavage for 14 days. At the end of the experiment, mice were euthanized by cervical dislocation, and kidneys and blood samples were collected.

### Renal function

Serum blood-urea-nitrogen (BUN) was quantified using a QuantiChrom Urea Assay Kit (BioAssay Systems, USA), according to the manufacturer’s guidelines and instructions.

### Histology and immunofluorescence

Kidneys were fixed in 4% paraformaldehyde for 24 h and then embedded in paraffin. Periodic Acid-Schiff (PAS) staining was performed to assess renal pathological damage. For immunofluorescence (IF) staining of kidney tissue paraffin sections, they were dewaxed and rehydrated using standard histological procedures. After blocking non-specific antigens with serum at room temperature for 30 min, specific primary antibodies Kidney Injury Molecule 1 (KIM-1) (1:200, R & D System, USA), Lotus Tetragonolobus Lectin (LTL) (1:100, Vector Laboratories, USA), α-Smooth Muscle Actin (α-SMA) (1:100, Abcam, UK) and collagen I (1:200, Servicebio, Wuhan) were incubated with the slides at 4 °C overnight. Fluorescent secondary antibodies were then used for IF labelling. The nuclei were stained with 4’,6-diamidino-2-phenylindole (DAPI, Servicebio, Wuhan). Ten random images were carefully quantified for staining on each slide. The data were then analyzed using Image Pro Plus software (Media Cybernetics, Rockville, MD, USA).

### Protein extraction and enzymolysis

According to published article [36], the protein concentration of the samples was estimated using the bicinchoninic acid protein assay. Sodium dodecyl sulfate (SDS) containing L3, and 1× cocktail containing ethylenediaminetetraacetic acid (EDTA) were added to the samples, followed by placing the mixtures on ice for 5 min and adding dithiothreitol (DTT). After grinding and centrifugation, the collected supernatants were combined with DTT and incubated at 56℃ for 1 h. Iodoacetamide (IAM) was then added and the mixtures were placed in a dark room for 45 min. Cold acetone was then added to the protein solution at a ratio of 1:5 at -20℃ for 30 min. After centrifugation, the precipitate was collected, air dried, and the appropriate amount of SDS without L3 was added to obtain the target protein solution. Protein quantification was performed using the Bradford assay. Subsequently, proteins from each sample were added to the enzyme solution with a trypsin to substrate protein ratio of 1:20 and incubated at 37 °C for 4 h. The resulting digested peptide solution was desalted and the peptide liquid obtained was freeze-dried.

### Protein peptide iTRAQ labeling and isolation

Sample peptides were labelled using the iTRAQ reagent kit. Each sample was run on a Shimadzu LC-20AD liquid phase system. A Gemini C18 column was used for liquid phase separation. Peptide samples were redissolved in mobile phase A (5% ACN, pH9.8) and eluted at a gradient of 4uL/min. The elution peak was then monitored at 214 nm, and the component was collected every 3.15 min. 20 components were obtained by combining them with a chromatographic elution peak diagram, and then frozen drained [36].

### Nano-HPLC-MS/MS analysis

The drained peptide samples were redissolved in mobile phase A (2%ACN, 0.1% FA), and the supernatant was separated using UltiMate 3000 UHPLC (Thermo Fisher Scientific, USA). Samples were first enriched and desalted in a trap column, and then separated in series on a self-loaded C18 column at a flow rate of 300 NL /min through an effective gradient. The separated peptide segments were ionized using the nanoESI source and then injected into the tandem mass spectrometer Q-Exactive HF X (Thermo Fisher Scientific, San Jose, CA) for detection in the Data Dependent Acquisition (DDA) mode [36].

### Database searching

The original mass spectrometry data were converted to MGF format using Proteome Discoverer software (version 1.4.0.288; Thermo Fisher Scientific). All MS/MS samples were analyzed using Mascot (version 2.3.02; Matrix Science, London, UK), which was used to search the NCBI database. In addition, the Mascot search selected Carbamidomethyl (C), iTRAQ8plex (N-terminal) and iTRAQ8plex (K) as fixed modifications, and Oxidation (M), Deamidated (NQ) and iTRAQ8plex (Y) as variable modifications.

### Quantitative data analysis

iTRAQ quantification was performed using IQuant software (https://analyticalsciencejournals.onlinelibrary.wiley.com/doi/full/10.1002/pmic.201300361) [37], which integrates the Mascot Percolator algorithm (https://linkinghub.elsevier.com/retrieve/pii/S1535947620326463) with a percolator algorithm with a false discovery rate of less than 1%. All unique peptides were quantified with at least one unique spectrum, and experimental biases were normalized with the weighted average. Statistical analysis was performed in the R environment (permutation tests, *P* < 0.05 was considered statistically significant). Differentially expressed proteins were selected when foldchange > 1.2 and Qvalue < 0.05 and then functionally assigned by the Cluster of Orthologous Groups of Proteins (COGs) and the Kyoto Encyclopedia of Genes and Genomes (KEGG) database.

## Results

### The L-*Lac.z* ameliorated BIR induced acute kidney injury

**Fig. 1:**
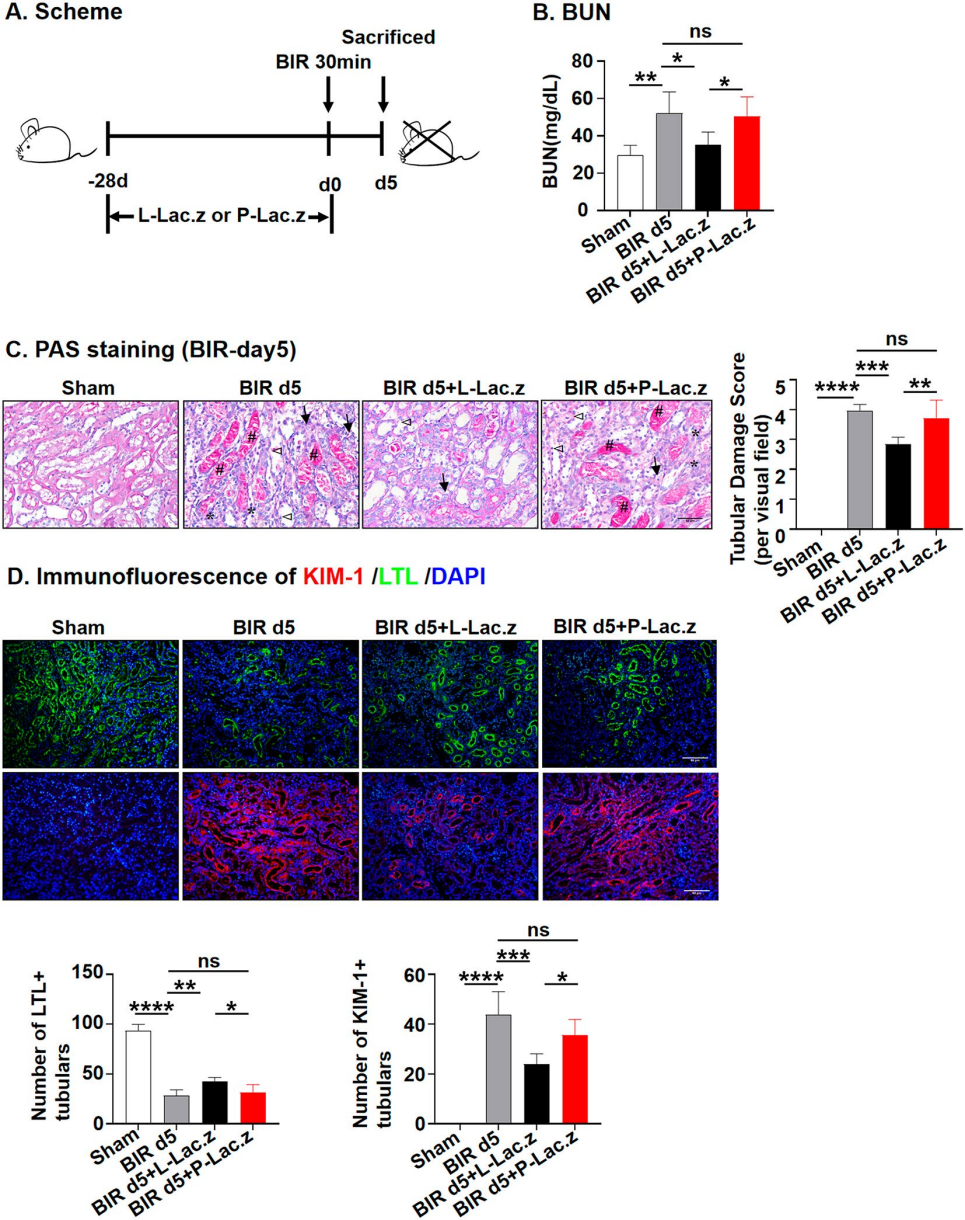
The effects of L-*Lac.z* and P-*Lac.z* on BIR-induced AKI. (**A**) Scheme of the experimental program: mice were pretreated with L-*Lac.z* or P-*Lac.z* by gavage for 28 days, and on day 28, mice were subjected to the BIR surgery, followed by no probiotic intervention, and were killed on day 5 after surgery. (**B**) Serum BUN levels. Serum BUN levels in the untreated and *Lac.z* pretreated groups on day 5 after BIR-induced renal injury. (**C**) PAS staining. Micrographs (40×) of the kidney PAS staining on day 5 after BIR-induced renal injury (left) and tubular injury score analysis (right) (*: necrosis; →: tubular dilation; #: casts formation; ◅: brush border loss). (**D**) Immunofluorescence. Representative photomicrographs (20×) of KIM-1 (red) and LTL (green) in the kidney on day 5 after BIR-induced renal injury and the number of LTL or KIM-1 positive tubules. ∗ *P* < 0.05, ∗∗*P* < 0.01, ∗∗∗*P* < 0.001 as determined by one-way ANOVA (**B**–**D**). Scale bar, 50 μm. Data represent mean ± SD, *n* = 5–6/group

To explore the potential protective effects of L-*Lac.z* and P-*Lac.z* against AKI, mice were pretreated with L-*Lac.z* or P-*Lac.z* (1 × 10^9 CFU per day) by gavage for 28 days, and on day 28, mice were subjected to the BIR surgery, followed by no probiotic intervention, and were killed on the fifth day after surgery following the procedure (Fig. 1A). Compared to the BIR group, pretreatment with L-*Lac.z* reduced the serum BUN levels (Fig. 1B) and resulted in less pathological damage, including necrosis, tubular dilation, casts formation, and brush border loss (Fig. 1C). These protective effects were not observed in the P-*Lac.z* group. Additionally, we operated immunostaining of LTL as a marker of normal proximal renal tubular and KIM-1 as a marker of proximal renal tubular injury during AKI, to further investigate these protective mechanisms. The results showed that KIM-1-positive tubules were significantly reduced, whereas LTL-positive tubules were significantly increased only in the L-*Lac.z* group (Fig. 1D). These results suggest that P-*Lac.z* pretreatment does not attenuate BIR-induced AKI, whereas L-*Lac.z* does.

### The L-*Lac.z* attenuated BIR induced chronic kidney fibrosis

**Fig. 2:**
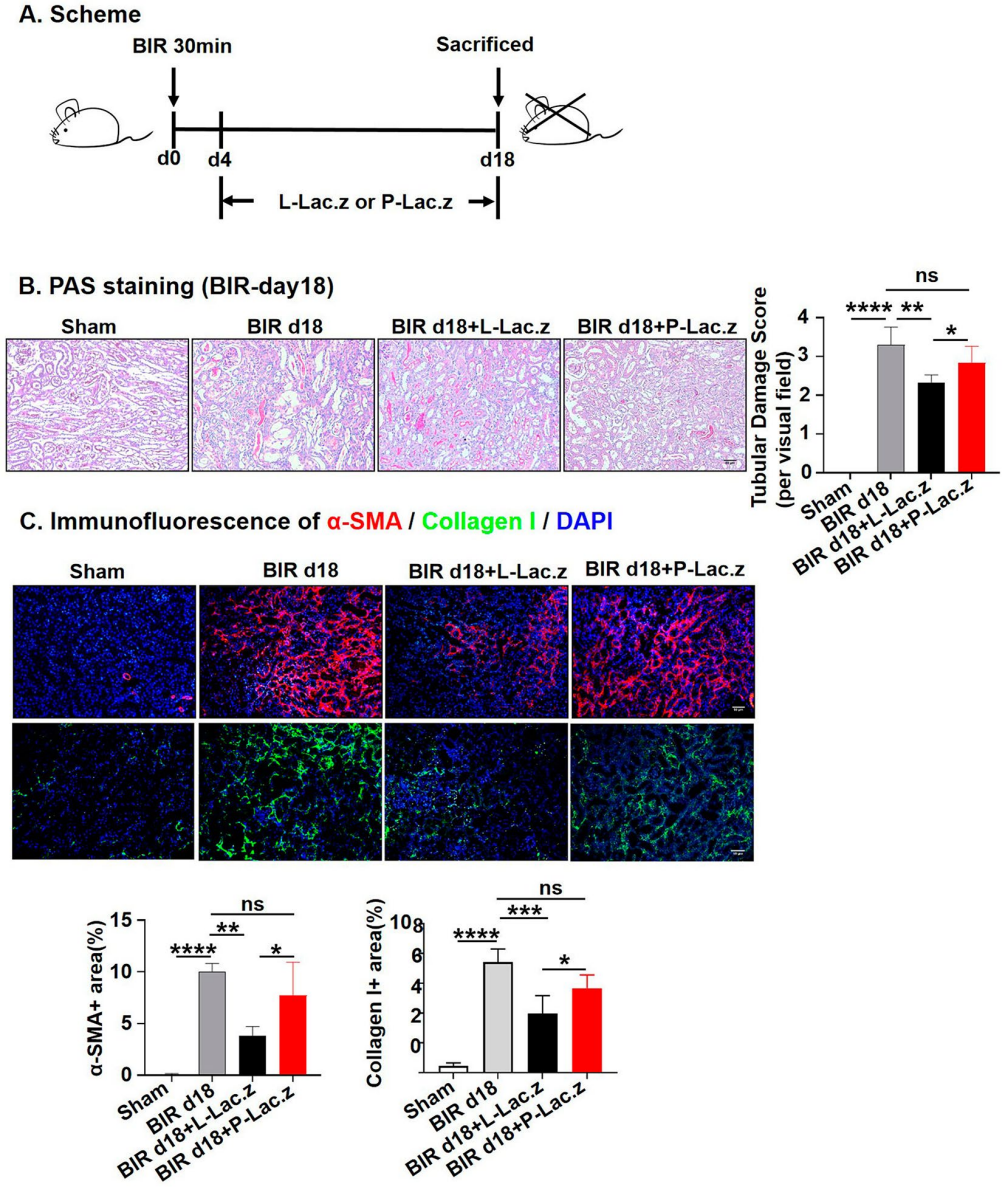
The effects of L-*Lac.z* and P-*Lac.z* on BIR-induced chronic kidney fibrosis. (**A**) Scheme of the experimental program: mice were subjected to the BIR surgery, and on day 4 after surgery, mice were treated with L-*Lac.z* or P-*Lac.z* by gavage for 14 days. (**B**) PAS staining. Micrographs (20×) of the kidney PAS staining on day 18 after BIR-induced renal injury (left) and the tubular damage score analysis (right). (**C**) Immunofluorescence. Representative photomicrographs (20×) of α-SMA (red) or collagen I (green) in the kidney at day 18 after BIR-induced renal injury and the percentage of α-SMA -positive or collagen I-positive area. ∗ *P* < 0.05, ∗∗*P* < 0.01, ∗∗∗*P* < 0.001 as determined by one-way ANOVA (**B**–**C**). Scale bar, 50 μm. Data represent mean ± SD, *n* = 3–5/group

Mice were orally gavaged with L-*Lac.z* or P-*Lac.z* for 14 days after renal BIR injury (Fig. 2A). The PAS staining showed that P-*Lac.z* couldn’t significantly alleviate the renal injury, while the L-*Lac.z* group showed reduced tubular dilatation, necrosis and casts formation, along with decreased glomerular atrophy and fibrous deposition compared to the BIR group (Fig. 2B). Immunofluorescence staining for α-SMA and collagen I, markers of fibrosis, showed that the percentage of α-SMA-positive or collagen I-positive area was remarkably lower (*P* < 0.05) in the L-*Lac.z* group, but not in the P-*Lac.z* group (Fig. 2C). These results indicated that whereas P-*Lac.z* pretreatment couldn’t delay BIR-induced chronic renal interstitial fibrosis, L-*Lac.z* treatment did.

### Differentially expressed proteins identified between L-*Lac.z* and P-*Lac.z*

To further investigate the potential mechanisms underlying the differential effects of L-*Lac.z* and P-*Lac.z*, the analysis of differentially expressed proteins between the two groups was performed. The results revealed that the expression of a total of 58 proteins of P-*Lac.z* significantly changed (> 2.0-folds, *P* < 0.05) compared to the initial live form, including 39 down-regulated proteins (Table 1) and 19 up-regulated proteins (Table 2). Many of these proteins could be categorized into specific COG functional categories.

**Table 1:** Down-regulated P-Lac.z proteins in comparison with the initial live form

Protein	Function	COG	Ratio	*P*-value
K6QG06_LACCA	PadR family transcriptional regulator	K	0.78	< 0.05
F5CPF9_LACCA	DNA-directed RNA polymerase subunit alpha	K	0.65	< 0.05
A0A0H0YRS1_LACCA	MurR/RpiR family transcriptional regulator	K	0.81	< 0.05
K6QLU8_LACCA	XRE family transcriptional regulator	K	0.70	< 0.05
A0A454Y0E5_LACCD	HTH-type transcriptional regulator	K	0.79	< 0.05
A0A1Q9MND5_LACCA	LacI family transcriptional regulator	K	0.82	< 0.05
A0A0E2BTC7_LACCA	peptide chain release factor 2	J	0.75	< 0.05
A0A1Q9MGF0_LACCA	amidase	J	0.69	< 0.05
K0N619_LACCA	pseudouridine synthase	J	0.83	< 0.05
A0A1Z2F4R4_LACCA	macro domain-containing protein	J	0.79	< 0.05
A0A0H0YL05_LACCA	6-phosphogluconate dehydrogenase	G	0.82	< 0.05
A0A1Q9MMN9_LACCA	alpha-glycosidase	G	0.71	< 0.05
A0A125UBS5_LACCD	PTS sorbitol transporter subunit IIB	G	0.78	< 0.05
A0A0E2BTP0_LACCA	Neopullulanase	G	0.81	< 0.05
A0A1Q9ME56_LACCA	NAD-dependent DNA ligase LigA	L	0.80	< 0.05
K6QVI9_LACCA	DNA damage-inducible protein DinG	L	0.79	< 0.05
A0A0R1FEN4_LACCA	Segregation and condensation protein A	L	0.79	< 0.05
A0A0E2C3V8_LACCA	restriction endonuclease	V	0.58	< 0.05
A0A0R1F1B5_LACCA	ABC-type multidrug transport system	V	0.71	< 0.05
A0A1Z2F492_LACCA	Multidrug ABC transporter permease	V	0.54	< 0.05
A0A1Q9MMC8_LACCA	extracellular matrix binding protein	D	0.82	< 0.05
A0A0H0YMH6_LACCA	Cell division protein FtsX	D	0.79	< 0.05
A0A0R1EZN5_LACCA	dTDP-glucose 4,6-dehydratase	M	0.82	< 0.05
A0A1Z2F3W1_LACCA	capsular biosynthesis protein	M	0.80	< 0.05
K6QU85_LACCA	NADH: flavin oxidoreductase	C	0.83	< 0.05
A0A0R1FBY5_LACCA	NADH-dependent flavin oxidoreductase	C	0.80	< 0.05
K6SR35_LACCA	vitamin-B12 independent methionine synthase	E	0.58	< 0.05
K6R597_LACCA	amino acid transporter	E	0.64	< 0.05
A0A0E2BP10_LACCA	GHKL domain-containing protein	T	0.80	< 0.05
K6QL41_LACCA	transposase	X	0.83	< 0.05
A0A125U7Y6_LACCD	sensor histidine kinase	VT	0.81	< 0.05
A0A0R1F898_LACCA	riboflavin biosynthesis protein RibF	H	0.76	< 0.05
A0A0R1F8G8_LACCA	signal recognition particle-docking protein FtsY	U	0.83	< 0.05
A0A0R1F4U5_LACCA	hypothetical protein	S	0.83	< 0.05
A0A0E2BVT5_LACCA	hypothetical protein	-	0.80	< 0.05
K6QTC4_LACCA	hypothetical protein	-	0.82	< 0.05
S6BR45_LACCA	hypothetical protein	-	0.60	< 0.05
A0A0R1F8U6_LACCA	hypothetical protein	-	0.81	< 0.05
A0A125YCB8_LACCD	hypothetical protein	-	0.72	< 0.05

**Table 2:** Up-regulated P-Lac.z proteins in comparison with the initial live form

Protein	Function	COG^a^	Ratio	*P*-value
A0A125UBX4_LACCD	sugar ABC transporter permease	G	1.52	< 0.05
A0A0E2BTQ9_LACCA	glucose-6-phosphate dehydrogenase	G	1.44	< 0.05
A0A0E2BP69_LACCA	beta-glucosidas	G	1.20	< 0.05
A0A0H0YL38_LACCA	pseudouridine synthase	J	1.32	< 0.05
A0A0R1EYI1_LACCA	Ribosomal RNA small subunit methyltransferase D	J	1.21	< 0.05
A0A0E2BQC0_LACCA	hypothetical protein	S	1.22	< 0.05
S6BP25_LACCA	Putative hydrolase	S	1.21	< 0.05
K0N5U2_LACCA	Shikimate dehydrogenase	E	1.85	< 0.05
A0A1Q9MPW2_LACCA	restriction endonuclease	V	1.43	< 0.05
A0A0E2C0R6_LACCA	ABC transporter ATP-binding protein	M	1.22	< 0.05
A0A0R1F3E4_LACCA	metal ABC transporter permease	P	1.42	< 0.05
A0A1Q9MMN4_LACCA	ArsR family transcriptional regulator	K	1.24	< 0.05
S6BVE1_LACCA	sensor histidine kinase	T	1.20	< 0.05
RECU_LACCB	Holliday junction resolvase RecU	R	1.21	< 0.05
A0A158SHI6_LACCD	esterase	Q	1.22	< 0.05
A0A0E2BQY0_LACCA	hypothetical protein	-	1.71	< 0.05
A0A125UBH4_LACCD	hypothetical protein	-	1.26	< 0.05
A0A0E2BUL6_LACCA	hypothetical protein	-	1.21	< 0.05
A0A1Z2EZX3_LACCA	hypothetical protein	-	1.24	< 0.05

^a^COG functional categories: [J], Translation, ribosomal structure and biogenesis; [G], Carbohydrate transport and metabolism; [E], Amino acid transport and metabolism; [T], Signal transduction mechanisms; [C], Energy production and conversion; [Q], Secondary metabolites biosynthesis, transport and catabolism; [M], Cell wall/membrane/envelope biogenesis; [R], General function prediction only; [S], Function unknown; [V], Defense mechanisms; [P], Inorganic ion transport and metabolism; [K], Transcription; [D], Cell cycle control, cell division, chromosome partitioning; [L], Replication, recombination and repair; [X], Mobilome: prophages, transposons; [VT], Defense mechanisms; Signal transduction mechanisms; [U], Intracellular trafficking, secretion, and vesicular transport

Among 39 down-regulated proteins, six were involved in transcription(K), including a PadR family transcriptional regulator (K6QG06_LACCA), a DNA-directed RNA polymerase subunit alpha (F5CPF9_LACCA), a MurR/RpiR family transcriptional regulator (A0A0H0YRS1_LACCA), a XRE family transcriptional regulator (K6QLU8_LACCA), an HTH-type transcriptional regulator (A0A454Y0E5_LACCD), and a LacI family transcriptional regulator (A0A1Q9MND5_LACCA) respectively. Four down-regulated proteins fell into translation, ribosomal structure and biogenesis(J), namely peptide chain release factor 2 (A0A0E2BTC7_LACCA), amidase (A0A1Q9MGF0_LACCA), pseudouridine synthase (K0N619_LACCA) and macro domain-containing protein (A0A1Z2F4R4_LACCA), respectively. Besides, some of the down-regulated proteins fell into the COG classes G, C and E, which are associated with metabolism and biosynthesis. These included the 6-phosphogluconate dehydrogenase (A0A0H0YL05_LACCA), alpha-glycosidase (A0A1Q9MMN9_LACCA), PTS sorbitol transporter subunit IIB (A0A125UBS5_LACCD), Neopullulanase (A0A0E2BTP0_LACCA), NADH: flavin oxidoreductase (K6QU85_LACCA) and NADH-dependent flavin oxidoreductase (A0A0R1FBY5_LACCA), vitamin-B12 independent methionine synthase (K6SR35_LACCA) and amino acid transporter (K6R597_LACCA). Three other down-regulated proteins fell into class L, including the NAD-dependent DNA ligase LigA (A0A1Q9ME56_LACCA), the DNA damage inducible protein DinG (K6QVI9_LACCA), the segregation and condensation protein A (A0A0R1FEN4_LACCA), which were associated with maintaining gene stability. Three down-regulated proteins related to defense mechanisms were restriction endonuclease (A0A0E2C3V8_LACCA) and ABC-type multidrug transport system (A0A0R1F1B5_LACCA, A0A1Z2F492_LACCA).

In contrast to the spectrum of down-regulated expressed proteins, 19 differentially expressed proteins were up-regulated. Three of them belonged to the COG class G: carbohydrate transport and metabolism, including a sugar ABC transporter permease (A0A125UBX4_LACCD), a glucose-6-phosphate dehydrogenase (A0A0E2BTQ9_LACCA) and a beta-glucosidase (A0A0E2BP69_LACCA). Two other up-regulated proteins were involved in translation, ribosomal structure and biogenesis (J), namely the pseudouridine synthase (A0A0H0YL38_LACCA) and the ribosomal RNA small subunit methyltransferase D (A0A0R1EYI1_LACCA). The functions of several other differentially expressed proteins were unknown.

## Discussion

In our study, we compared the effects of L-*Lac.z* and P-*Lac.z* against AKI and subsequent chronic renal fibrosis. Consistent with our previous research [30], the L-*Lac.z* demonstrated the ability to alleviate BIR-induced AKI and chronic kidney fibrosis, as evidenced by a decrease in serum BUN, less pathological damage and reduced interstitial fibrosis, while these effects were not prominently observed in the P-*Lac.z* group. The subsequent proteomic results unveiled that 39 proteins were down-regulated and 19 proteins were up-regulated in P-*Lac.z* compared to the live form. Among these down-regulated proteins, the majority were involved in translation, ribosomal structure and biogenesis (A0A0E2BTC7_LACCA, A0A1Q9MGF0_LACCA, K0N619_LACCA, A0A1Z2F4R4_LACCA), carbohydrate transport and metabolism (A0A0H0YL05_LACCA, A0A1Q9MMN9_LACCA, A0A125UBS5_LACCD, A0A0E2BTP0_LACCA), defense mechanisms (A0A0E2C3V8_LACCA, A0A0R1F1B5_LACCA, A0A1Z2F492_LACCA), transcription (K6QG06_LACCA, F5CPF9_LACCA, A0A0H0YRS1_LACCA, K6QLU8_LACCA, A0A454Y0E5_LACCD, A0A1Q9MND5_LACCA), replication, recombination, and repair (A0A1Q9ME56_LACCA; K6QVI9_LACCA, A0A0R1FEN4_LACCA), while the few up-regulated proteins were involved in translation, ribosomal structure and biogenesis (A0A0H0YL38_LACCA, A0A0R1EYI1_LACCA), carbohydrate transport and metabolism (A0A125UBX4_LACCD, A0A0E2BTQ9_LACCA, A0A0E2BP69_LACCA). These findings suggested that the pasteurized bacteria might have a reduced ability to use nutrients, and also might be less resistant to stress resistance and defense in the higher temperature on environment of pasteurization, which suggested some importance of these physiological activities in environments on which a thermal stress like that of the pasteurization occurred. Previous studies implied that the cells of *Lac.z* at stationary phase were tolerant to some stresses including acid (pH 2.5), or heating at 56 °C for 1 h, and the optimal growth temperature of *Lac.z* is 37℃, so we supposed that the heat stress (37 °C) in the gut has little effect on L-*Lac.z*.

Research has shown that certain probiotics, such as *Akkermansia muciniphila* [38, 39], *Lactobacillus rhamnosus ATCC 7469* [32] and *Lactobacillus plantarum* [40, 41], both in their active form and after pasteurization, may have a protective role in certain diseases [39, 42, 43]. For example, although *Akkermansia muciniphila* was killed by pasteurization operation, Amuc_1100, a specific protein enriched in its outer membrane could withstand pasteurization and improve the intestinal barrier by interacting with Toll-like receptor2, partially preserving the beneficial effects of this bacterium [39]. In our proteomic analysis, we observed that after pasteurization, only the expression of proteins related to growth and stress resistance were significantly altered, with no detection of other important proteins that could direct contact with the host, such as outer membrane proteins or surface piliation, thus the role of *Lac.z* in exerting nephroprotective effects may be more dependent on the vital physiological activities of living bacteria such as metabolism or interacting with other gut microbiota to alter gut flora composition, as demonstrated in our previously published paper: L-*Lac.z* exerted renoprotective effects by alleviating gut dysbiosis, increasing levels of beneficial gut metabolites, such as SCFAs and niacinamide [18].

There are some limitations to our study. firstly, in the treatment of the intervention AKI model, mice were pretreated with live or pasteurized *Lac.z* by gavage for 28 days, and this time period was in reference to our previously published article [30], and we don’t provide any information about the effect of this time period on the restoration of physiological activities by the pasteurized *Lac.z*. Secondly, our proteomic results were based on the consequence of the altered protein expression (down- and up-regulated proteins) due to the pasteurization procedure, but there may be other factors which could influence our results, such as protein composition and the formation of new protein aggregates due to the direct effects of temperature.

In conclusion, our research suggested that L-*Lac.z* attenuated AKI and subsequent chronic renal fibrosis, whereas these effects were eliminated after pasteurization. The specific nephroprotective effects of L-*Lac.z* may be independent of the interaction of live probiotics with the host.

## Electronic supplementary material

Below is the link to the electronic supplementary material.

Supplementary Material 1

## Data Availability

Data for this study can be reasonably obtained from the corresponding authors upon request.
