# Supplementary figures and images for: Effects of live and pasteurized forms of Lactobacillus casei Zhang on acute kidney injury and chronic renal fibrosis
Source: Braz J Microbiol. 2024 Aug 26;55(4):3699–709. doi: 10.1007/s42770-024-01491-y (PMC11712044; doi:10.1007/s42770-024-01491-y)

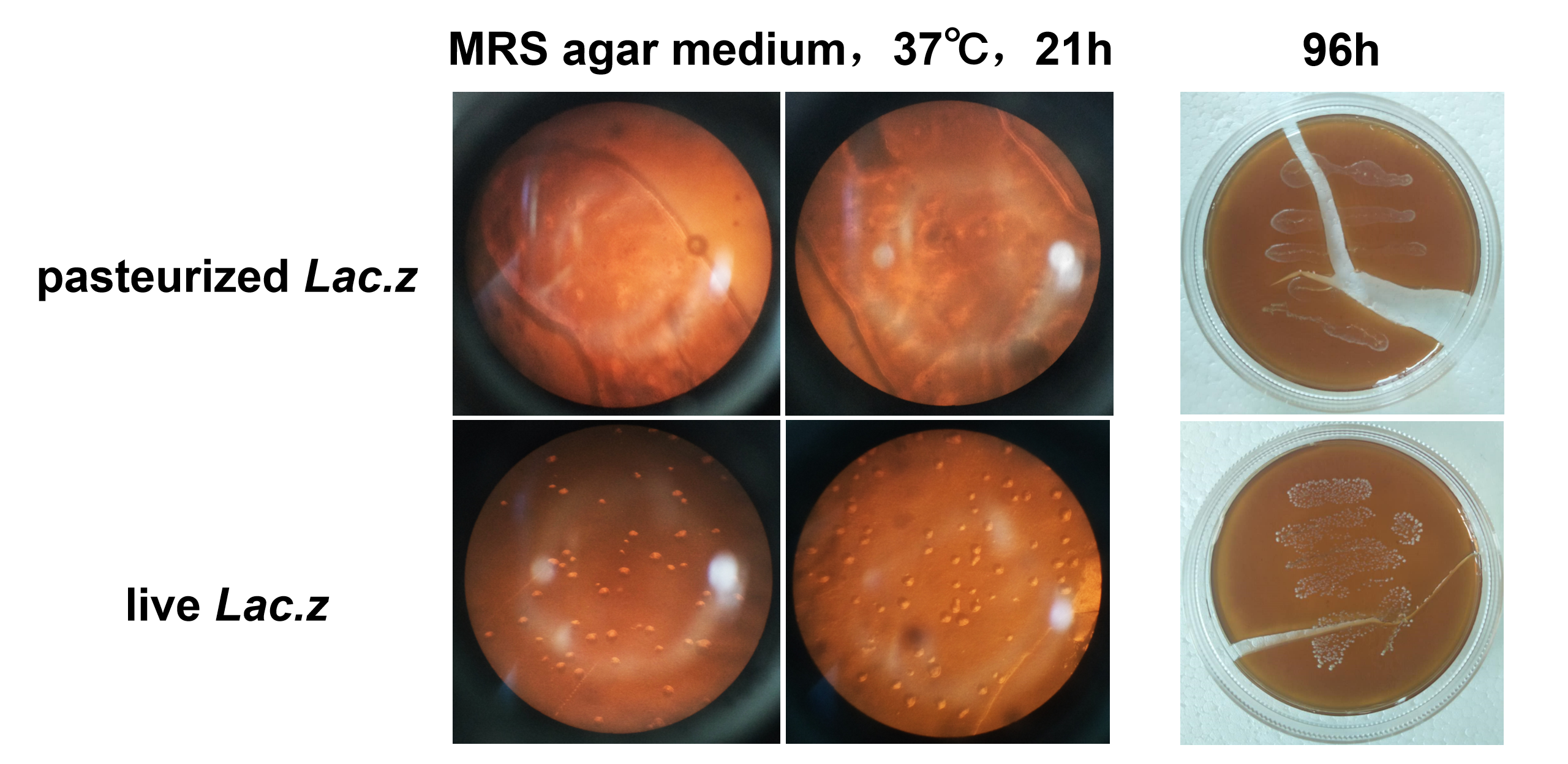

Supplement: Supplementary file 1 — Supplementary Material 1 [file 42770_2024_1491_MOESM1_ESM.tif]
